# Supplementary figures and images for: Comparison of efficacy and costs between robotic-assisted and conventional thoracoscopic approaches for partial pulmonary resection: a systematic review and meta-analysis of propensity score-matched studies
Source: PeerJ. 2025 Aug 29;13:e19911. doi: 10.7717/peerj.19911 (PMC12401028; doi:10.7717/peerj.19911)

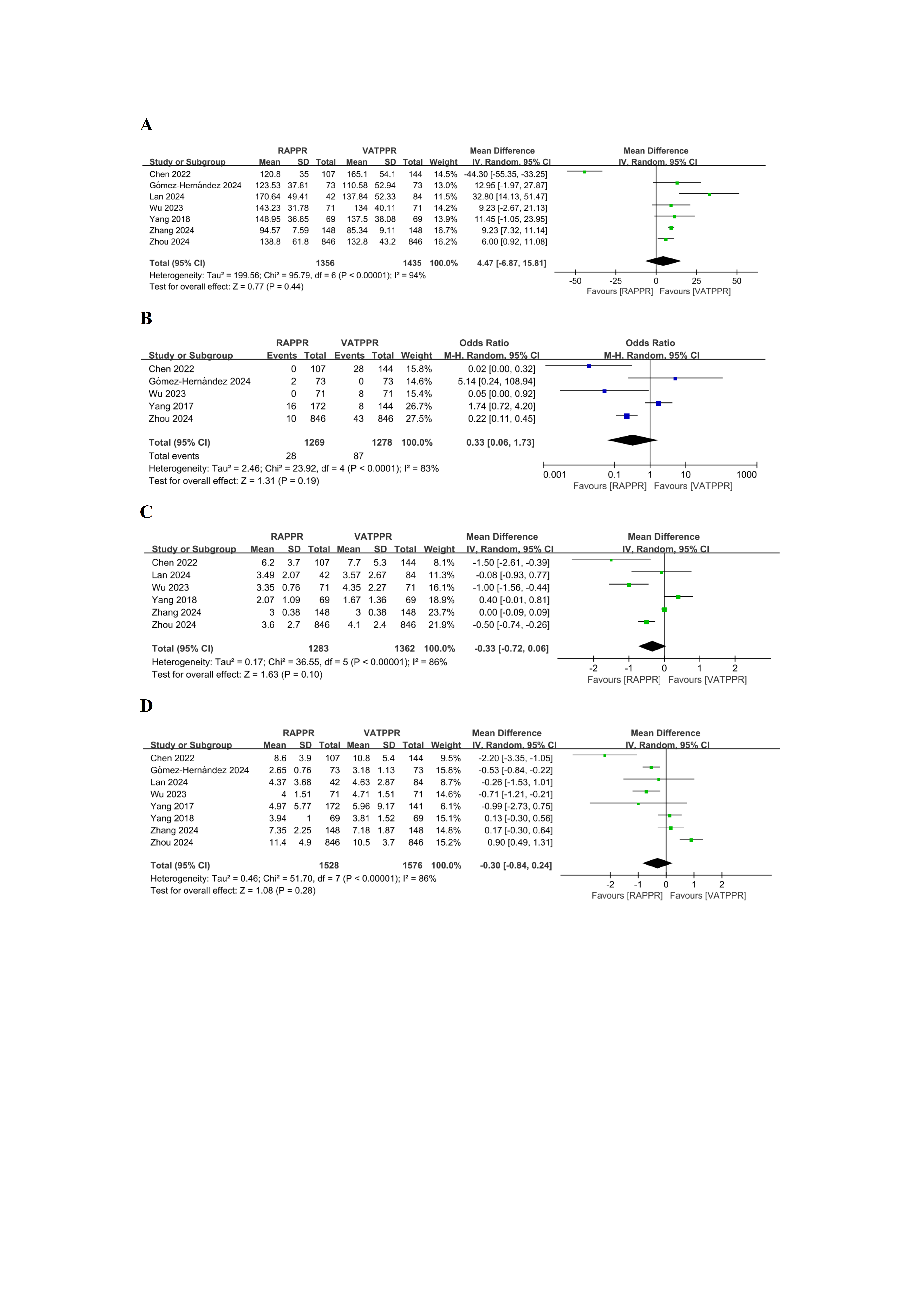

Supplement: Supplemental Information 2 [file peerj-13-19911-s002.png]
